# Preoperative differentiation of hepatocellular carcinoma with peripheral rim-like enhancement from intrahepatic mass-forming cholangiocarcinoma on contrast-enhanced MRI

**DOI:** 10.3389/fonc.2022.986713

**Published:** 2022-11-23

**Authors:** Sisi Zhang, Lei Huo, Yayuan Feng, Juan Zhang, Yuxian Wu, Yiping Liu, Lun Lu, Ningyang Jia, Wanmin Liu

**Affiliations:** ^1^ Department of Radiology, Shanghai Eastern Hepatobiliary Surgery Hospital, The Third Affiliated Hospital of Naval Medical University, Shanghai, China; ^2^ Department of Radiology, Tongji Hospital, School of Medicine, Tongji University, Shanghai, China

**Keywords:** magnetic resonance imaging, hepatocellular carcinoma, liver cancer, intrahepatic cholangiocarcinoma, differential diagnosis

## Abstract

**Purpose:**

The present study aimed to determine the reliable imaging features to distinguish atypical hepatocellular carcinoma (HCC) with peripheral rim-like enhancement from intrahepatic mass-forming cholangiocarcinoma (IMCC) on contrast-enhanced magnetic resonance imaging (MRI).

**Methods:**

A total of 168 patients (130 male, 57.10 ± 10.53 years) pathological confirmed HCC or IMCC who underwent contrast-enhanced MRI between July 2019 and February 2022 were retrospectively included. Univariate and multivariate logistic regression analyses were used to determine independent differential factors for distinguishing HCC from IMCC, and the model was established. Bootstrap resampling 1000 times was used to verify the model, which was visualized by nomograms. The predictive performance of the model was evaluated based on discrimination, calibration, and clinical utility.

**Results:**

Radiological capsule (OR 0.024, 95% CI: 0.006, 0.095, P<0.001), heterogeneous signal intensity (SI) on T1WI (OR 0.009, 95%CI: 0.001,0.056, P<0.001) were independent differential factors for predicting HCC over IMCC. A lobulated contour (OR 11.732, 95%CI: 2.928,47.007, P = 0.001), target sign on DP (OR 14.269, 95%CI: 2.849,82.106, P = 0.007), bile duct dilatation (OR 12.856, 95%CI: 2.013, P = 0.001) were independent differential factors for predicting IMCCs over HCCs. The independent differential factors constituted a model to distinguish atypical HCCs and IMCCs. The area under receiver operating characteristic (ROC) curve, sensitivity, and specificity values of the model were 0.964(0.940,0.987), 0.88, and 0.906, indicating that the model had an excellent differential diagnostic performance. The decision curve analysis (DCA) curve showed that the model obtained a better net clinical benefit.

**Conclusion:**

The present study identified reliable imaging features for distinguishing atypical HCCs with peripheral rim-like enhancement from IMCCs on contrast-enhanced MRI. Our findings may help radiologists provide clinicians with more accurate preoperative imaging diagnoses to select appropriate treatment options.

## 1 Introduction

Hepatocellular carcinoma (HCC) is the sixth most common neoplasm and the third leading cause of death from cancer worldwide (1, 2). HCC is most commonly caused by cirrhosis, with 2-8% of cirrhosis cases developing into HCC each year (3–5). Intrahepatic cholangiocarcinoma (iCCA) is the second most common primary liver malignancy, and its worldwide incidence is increasing (6, 7). As a malignancy, iCCA often shares some common risk factors and clinical manifestations with HCC, which poses a challenge for the differential diagnosis of iCCA and HCC (8, 9). Therefore, it is necessary to find an effective and specific method for the differential diagnosis of HCC and iCCA.

Magnetic resonance imaging (MRI) is widely used for preoperative diagnoses and evaluation of liver tumors (10). HCC can be diagnosed with typical imaging characteristics of hyperenhancement in arterial phase and washout on portal or delayed phase images (10–13), which represent the characteristic vascular profile of HCC on dynamic contrast-enhanced magnetic resonance imaging (13). However, around 40% of HCCs do not show typical imaging features, and they may exhibit arterial phase hypovascularity or peripheral rim-like enhancement (14, 15).

Currently, these HCCs with atypical imaging features pose a significant diagnostic challenge to radiologists. In particular, HCC is difficult to differentiate from intrahepatic mass-forming cholangiocarcinoma (IMCC) if it presents with peripheral rim-like enhancement in the arterial phase followed by gradual filling of the contrast media (16, 17). Several studies have examined the imaging features of atypical HCC (16, 18), although studies focusing on the differentiation between HCC with peripheral rim-like enhancement and IMCC are rare. However, the distinction between HCCs and IMCCs is crucial for clinicians because they have significant differences in prognosis and treatment options (19). Therefore, it is essential to understand and identify reliable imaging features to help accurately differentiate atypical HCCs with peripheral rim-like enhancement from IMCCs.

Accordingly, the purpose of the present study was to determine the reliable imaging features and establish the optimal model to distinguish atypical HCCs with peripheral rim-like enhancement from IMCCs on contrast-enhanced MRI. In order to provide clinicians with more accurate preoperative imaging diagnoses to select appropriate treatment options.

## 2 Materials and methods

### 2.1 Patients

This study was approved by the ethics committee of eastern hepatobiliary surgery hospital, the third affiliated hospital of Shanghai naval military medical university, China, and waived the requirement of obtaining written informed consent.

**Figure 1:**
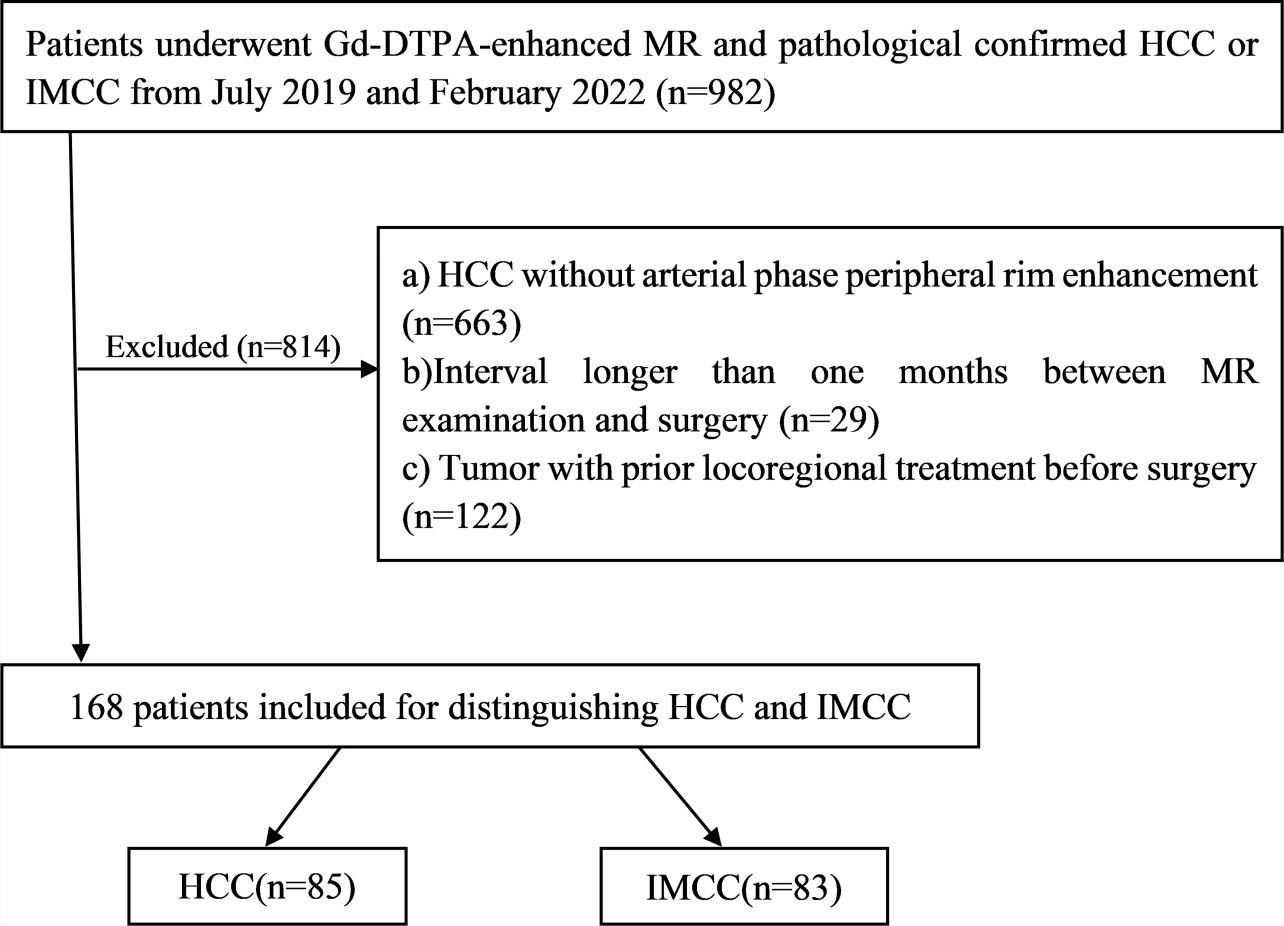
The workflow of patient selection for this study.

Between July 2019 and February 2022, a total of 168 patients (130 male, 57.10 ± 10.53 years) pathological confirmed HCCs or IMCCs, including 85 patients who were HCCs (74 male, 59.33 ± 10.79 years) and 83 patients were IMCCs (56 male, 54.82 ± 9.81 years) after preoperative Gd-DTPA-enhanced MRI met the following inclusion criteria (**Figure 1**): (a) complete histopathologic description of HCCs or IMCCs; (b) dynamic contrast-enhanced liver MR examination was performed within one month before operation, including complete scanning phase images (arterial phase, portal phase, delayed phase); (c) HCCs show atypical imaging features of peripheral rim-like enhancement in dynamic contrast-enhanced liver MRI, and (d) no locoregional treatment for tumor before MR examination.

### 2.2 Histopathology characteristics

A consensus of two experienced pathologists assessed the histopathological characteristics. All pathological sections were reviewed according to 2019 WHO classification standard (20), and the classification diagnosis of HCC and IMCC was performed. For HCC, histopathological factors for tumors were assessed: gross type, histological type, cell type, nuclear grade, fibrous capsule formation, vascular invasion, bile duct invasion, necrosis or hemorrhage. The nuclear grading scheme proposed by Edmondson-Steiner classified HCC tumors into four grades: I, II, III, and IV (21). For IMCC, the assessment of histopathological factors was evaluated as for HCC.

### 2.3 MRI examination

MR images were acquired using a GE Optima MR360 1.5T (Optima MR360, GE Healthcare, USA) equipped with an eight-channel abdominal coil. Patients fasted for four hours before the scan. Baseline MRI included T1-weighted turbo field-echo in-phase and opposed sequence (T1WI), Fat -suppressed T2-weighted images (Fs-T2WI). Diffusion-weighted imaging (DWI) was obtained by respiratory-triggered single-shot echo with b-values of 0 and 600 s/mm^2^.

Gadolinium meglumine acid (Gd‐DTPA) with a total dose of 0.1 mmoL/kg was injected into the median cubitus vein at a rate of 2.0 mL/s with a high-pressure syringe washing with 20 mL of normal saline. The arterial phase (AP), portal venous phase (PVP), and delay phase (DP) scans were performed 20–30 s, 50–60 s, and 90–120 s after the injection of Gd‐DTPA, respectively. Detailed scanner and scan parameters can be found in **Supplementary Table 1**.

### 2.4 MR imaging analysis

MR imaging analysis was performed by two radiologists (with more than 10 years of abdominal imaging experience) blinded to the histopathology information. Two radiologists independently evaluated imaging features. And inter-observer agreement was used to assess the consistency of the observed imaging features, variables with kappa value < 0.75 were removed. Inter-observer variability for each imaging feature can be found in **Supplementary Table 2**. Then, if their opinions were not consistent, a consensus decision was made after discussion among three radiologists. The case was included in the study only when the results of the two radiologists’ assessments were consistent.

The general imaging features were evaluated as follows: (a) tumor size was measured by selecting the length and diameter of the largest plane according to the liver imaging reporting and data system 2018 standard (22), including the mass capsule, when the mass was shown most clearly in the MRI enhanced portal phase images; (b) shape (round, lobulated or ill-defined); (c) margin, smooth edges (nodular tumors with smooth edges) and non-smooth edges (budding processes on cross-sectional and coronal images (23); (d) signal intensity (SI) on T1WI and T2WI (homogeneous or heterogeneous).

The enhanced imaging features were evaluated as follows: (a) enhancement of the tumor on AP, peripheral thin or thick rim enhancement (<30%,30–50%hyperintensity area of the tumor surface); (b) signal intensity of the tumor relative to liver parenchyma on PVP and DP; (c) gradual enhancement during dynamic contrast-enhanced phases.

The ancillary features were evaluated as follows: (a) bile duct dilation peripheral to tumor; (b) hepatic surface retraction at tumor attachment; (c) radiological capsule (partial or complete peripheral rim-like enhancement around the tumor on PVP or DP; (d) T2 central brightness (markedly higher than SI of spleen and tumor periphery) (24); (e) T2 central darkness (lower than liver SI) (25); (f) target sign (peripheral hyperintensity compared to central portion) on DWI (b = 600s/mm^2^); (g) intralesional fat; (h) portal vein embolus; (i) lymph node enlargement.

### 2.5 Statistical analysis

IBM SPSS Statistics (version 25; IBM) or R (version 3.6.0; http://www.r-project.org) were used for statistical analyses. Continuous variables conforming to the normal distribution and homogeneity of variance were represented as the means ± standard deviations and were compared using the Student’s t-test. Interobserver agreement between two radiologists were compared with the Kappa test. Inconsistent continuous variables were represented using the median (range) and compared with the Mann-Whitney U test. Categorical variables were compared using χ 2 test or Fisher’s exact test. The factors with P<0.05 in the univariate logistic regression analysis were included in multivariate logistic regression analysis (forward LR) to identify independent differential factors for distinguishing HCCs and IMCCs.

### 2.6 Model development and validation, and evaluation

Univariate and multivariate logistic regression (forward LR) were performed to determine the independent differential factors between HCCs and IMCCs. Combining these independent differential factors established the model for discriminating HCCs from IMCCs. Bootstrap resampling 1000 times was used to verify the model, which was visualized by nomograms (26). The differential diagnosis performance of the model was evaluated based on discrimination, calibration, and clinical utility. The discrimination for the model was quantified using the area under the receiver operating characteristic (ROC) curve, sensitivity, and specificity. The calibration curve analysis was performed to evaluate the consistency between the tumor types discriminated by the model and the actual tumor types. Decision curve analysis (DCA) was conducted to determine the clinical utility of the model by quantifying the net benefits at different threshold probabilities.

## 3 Results

### 3.1 Demographic and pathological characteristics of HCCs and IMCCs

Among the 168 patients, 85 (74 male, 59.33 ± 10.79 years) were HCC and 83 (56 male, 54.82 ± 9.81 years) were IMCC. The pathological and demographic characteristics of HCCs and IMCCs are shown in **Table 1**. Patients with HCCs were significantly older than those with IMCCs (59.33 ± 10.79 years vs. 54.82 ± 9.81years, P = 0.004). Sex was significantly differently in two groups (87.1% male for HCC vs. 67.5% male for IMCC, P = 0.003). Capsule formation (95.3% vs. 3.6%, P<0.001) and microscopic cirrhosis (50.6% vs. 13.3%, P<0.001) were more frequently present in HCCs than IMCCs.

**Table 1:** Demographic and pathological characteristics of HCCs and IMCCs.

Characteristic	HCCs (n=85)	IMCCs (n=83)	P value
Age (y)	59.33 ± 10.79	54.82 ± 9.81	0.004
Sex			
Male	74 (87.1%)	56 (67.5%)	0.003
Female	11 (12.9%)	27 (32.5%)	
HBV/HCV	78 (91.8%)	29 (34.9%)	<0.001
Edmondson-Steiner grade			
I- II	9 (16.5%)	/	/
III-IV	76 (89.4%)	/	
Capsule formation			
Absent	4 (4.7%)	80 (96.4%)	<0.001
Complete	21 (24.7%)	0	
Partial	60 (70.6%)	3 (3.6%)	
Microscopic cirrhosis			
Absent	42 (49.4%)	72 (86.7%)	<0.001
Present	43 (50.6%)	11 (13.3%)	

Data are numbers of patients with percentage in parentheses.

HCC, hepatocellular carcinoma; IMCC, intrahepatic mass-forming cholangiocarcinoma; HBV, hepatitis B virus; HCV, hepatitis C virus.

### 3.2 MRI characteristics of HCCs and IMCCs

#### 3.2.1 General MRI features

Tumor size, shape, margin, and SI on T1WI or T2WI were significantly different in the two groups. Tumor size of HCCs (7.8cm (5.8,10.2)) was larger than IMCCs size (5.6cm (4.1,7.0)) (P<0.001). A round contour (P<0.001), smooth margin (P = 0.060), and heterogeneous SI on T1WI or T2WI (P<0.001) were more commonly found in HCCs. A lobulated contour (P<0.001), non-smooth margin (P = 0.060), and homogeneous SI on T1WI or T2WI (P<0.001) were more commonly found in IMCCs. Evaluated characteristics for distinguishing HCCs and IMCCs are shown in **Table 2**.

**Table 2:** MRI characteristics of HCCs and IMCCs.

Characteristic	HCCs (n=85)	IMCCs (n=83)	P value
**Gradual MRI features**			
Tumor size(cm)	7.8 (5.8,10.2)	5.6 (4.1,7.0)	<0.001
Shape			
Round	68 (80.0%)	42 (50.6%)	<0.001
Lobulated/ill-defined	17 (20.0%)	41 (49.4%)	
Margin			
Smooth	40 (47.1%)	27 (32.5%)	0.060
Non-smooth	45 (52.9%)	56 (67.5%)	
SI on T1WI			
Homogeneous	35 (41.2%)	73 (88.0%)	<0.001
Heterogeneous	50 (58.8)	10 (12.0%)	
SI on T2WI			
Homogeneous	21 (24.7%)	51 (61.4%)	<0.001
Heterogeneous	64 (75.3%)	32 (38.6%)	
**Enhancement MRI features**			
AP enhancement			
Thick rim	36 (42.4%)	11 (13.3%)	<0.001
Thin rim	49 (57.6%)	72 (86.7%)	
SI on PVP			
Hypo	48 (56.5%)	53 (63.9%)	0.348
Iso/Hyper	37 (43.5%)	30 (36.1%)	
SI on DP			
Diffusely low	27 (31.8%)	8 (9.6%)	0.002
Target	53 (62.4%)	68 (81.9%)	
Iso	5 (5.9%)	7 (8.4%)	
Gradual enhancement	22 (25.9%)	58 (69.9%)	<0.001
**Ancillary features**			
Surface retraction			
Absent	81(95.3%)	62 (74.7%)	<0.001
Present	4(4.7%)	21 (25.3)	
Bile duct dilation			
Absent	81 (95.3%)	56 (67.5%)	<0.001
Present	4 (4.7%)	27 (32.5%)	
Radiological capsule			
Absent	16 (18.8%)	69 (83.1%)	<0.001
Present	69 (81.2%)	14 (16.9%)	
Intralesional fat			
Absent	68 (80.0%)	81 (97.6%)	<0.001
Present	17 (20.0%)	2 (2.4%)	
Central brightness on T2WI			
Absent	46 (54.1%)	68 (81.9%)	<0.001
Present	39 (45.9%)	15 (18.1%)	
Central darkness on T2WI			
Absent	61 (71.8%)	64 (77.1%)	0.482
Present	24 (28.2%)	19 (22.9%)	
Target sign on DWI			
Absent	43 (50.6%)	26 (31.3%)	0.013
Present	42 (49.4%)	57 (68.7%)	
Portal vein embolus			
Absent	65 (76.5%)	83 (100%)	<0.001
Present	20 (23.5%)	0	
Lymph node enlargement			
Absent	75 (88.2%)	57 (68.7%)	
Present	10 (11.8%)	26 (31.3%)	0.002

Data are numbers of patients with percentage in parentheses.

HCC, hepatocellular carcinoma; IMCC, intrahepatic mass-forming cholangiocarcinoma; SI signal intensity; AP, atrial phase; PVP, portal venous phase; DP, delayed phase; T2WI, T2-weighted image; DWI, diffusion-weighted imaging.

#### 3.2.2 Enhanced MRI features

HCCs more frequently showed a thick rim on AP images (P<0.001) and diffusely low SI on DP images (P = 0.002). IMCCs more frequently showed a thin rim on AP images (P<0.001), target sign on DP images (P = 0.002), and gradual enhancement (P<0.001).

#### 3.2.3 Ancillary features

**Figure 2:**
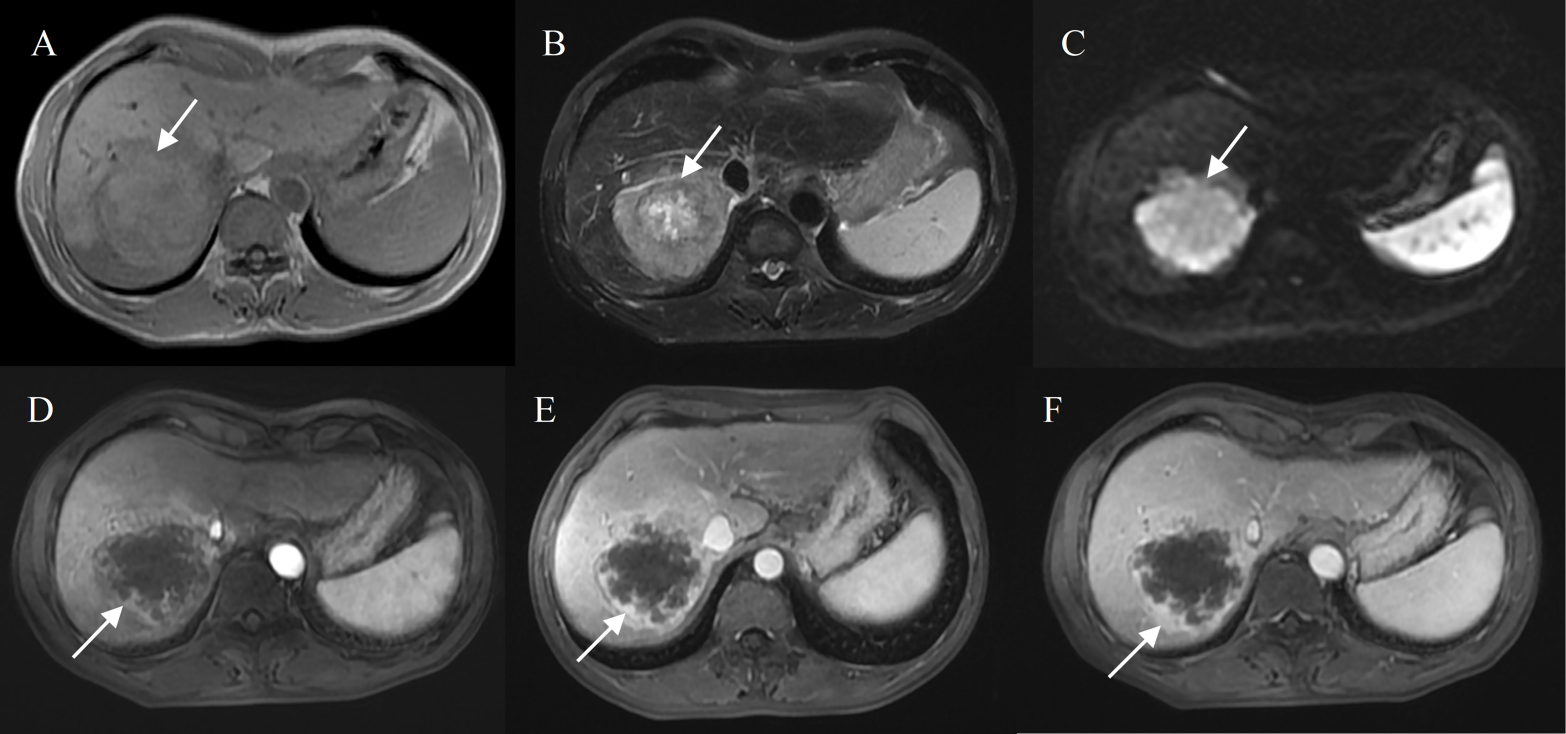
Hepatocellular carcinoma with peripheral rim enhancement in a 60-year-old male without chronic viral hepatitis. Gd-DTPA-enhanced MRI detected a round and non-smooth tumor (10.2 cm) with heterogeneous SI on T1WI and T2WI **(A, B)**, restricted diffusion **(C)**. Peripheral rim-like enhancement in the arterial phase **(D)** and sustained rim enhancement in the portal and delayed phase **(E, F)**. Incomplete capsule enhancement on portal venous phase **(E)** and delayed phase images **(F)**.

**Figure 3:**
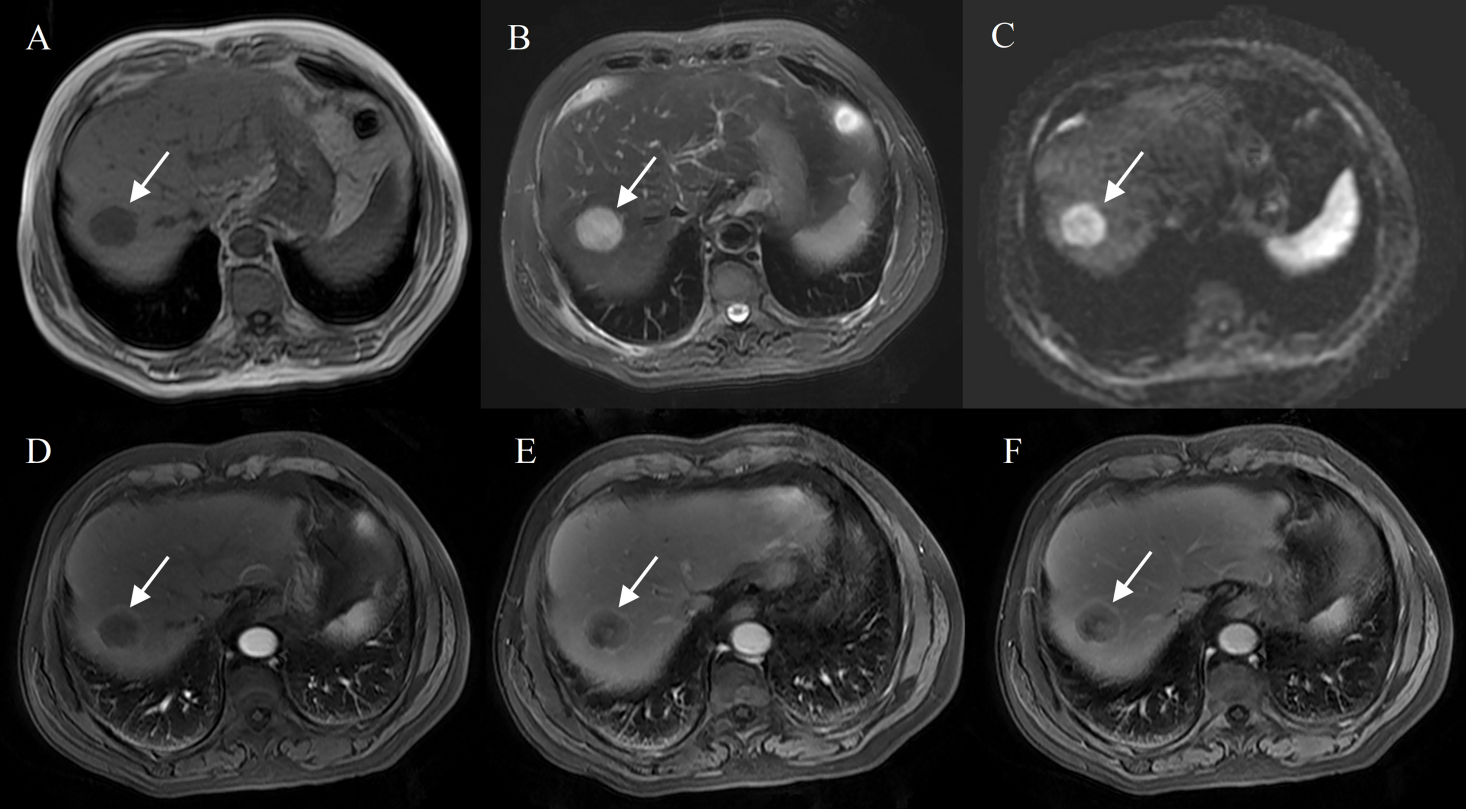
Hepatocellular carcinoma with peripheral rim enhancement in a 70-year-old male with hepatitis B virus. Gd-DTPA-enhanced MRI detected a round and smooth tumor (4.8 cm) with heterogeneous SI on T1WI and T2WI **(A, B)**, restricted diffusion **(C)**. Peripheral rim-like enhancement in the arterial phase **(D)**, complete capsule enhancement on portal venous phase **(E)** and delayed phase images **(F)**.

**Figure 4:**
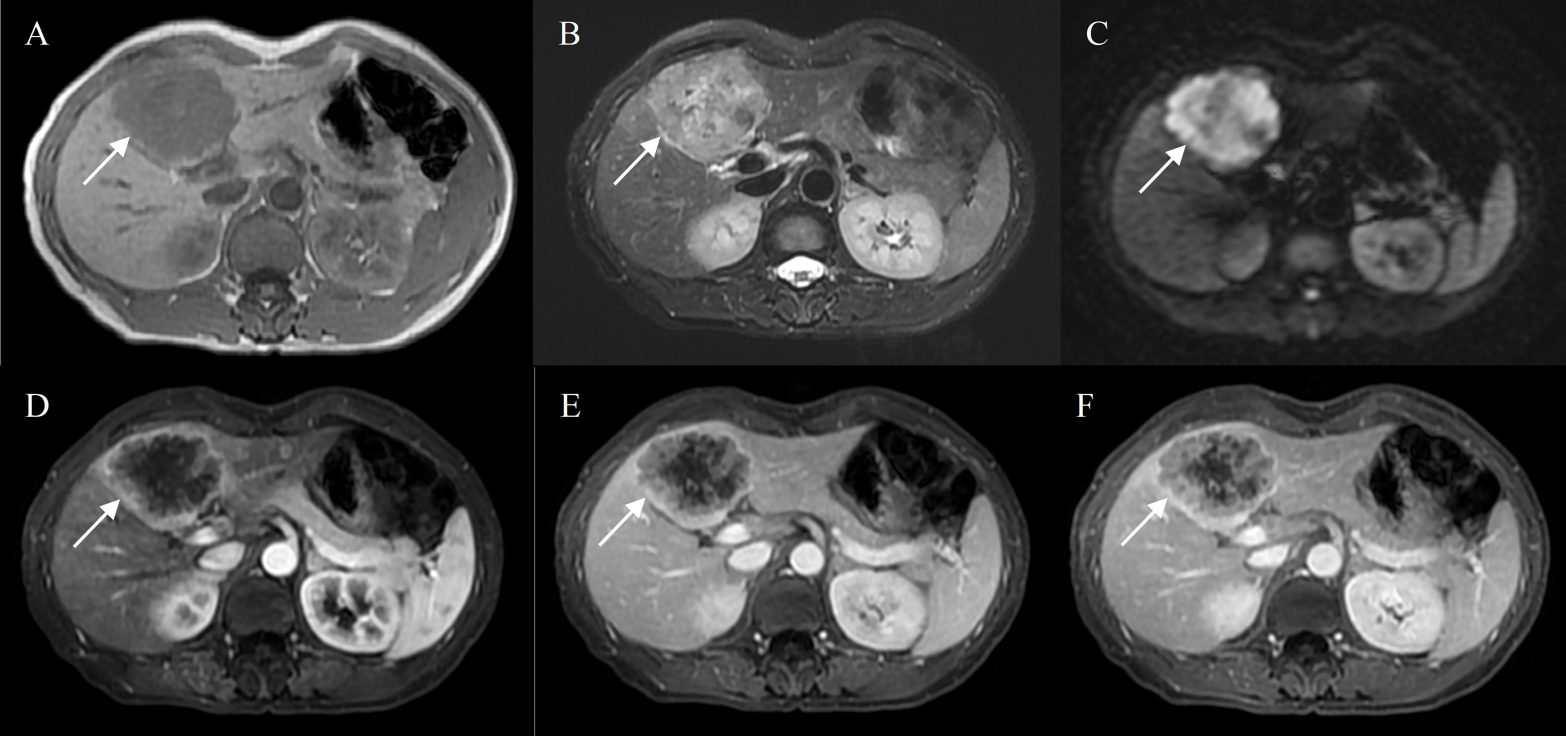
Intrahepatic mass-forming cholangiocarcinoma in a 50-year-old female with hepatitis B virus. Gd-DTPA-enhanced MRI detected a lobulated tumor (7.0 cm) with heterogeneous SI on T1WI and T2WI **(A, B)**, restricted diffusion **(C)**. Peripheral rim-like enhancement in the arterial phase **(D)**, followed by progressive filling of the contrast material in the portal and delayed phase **(E, F)**.

**Figure 5:**
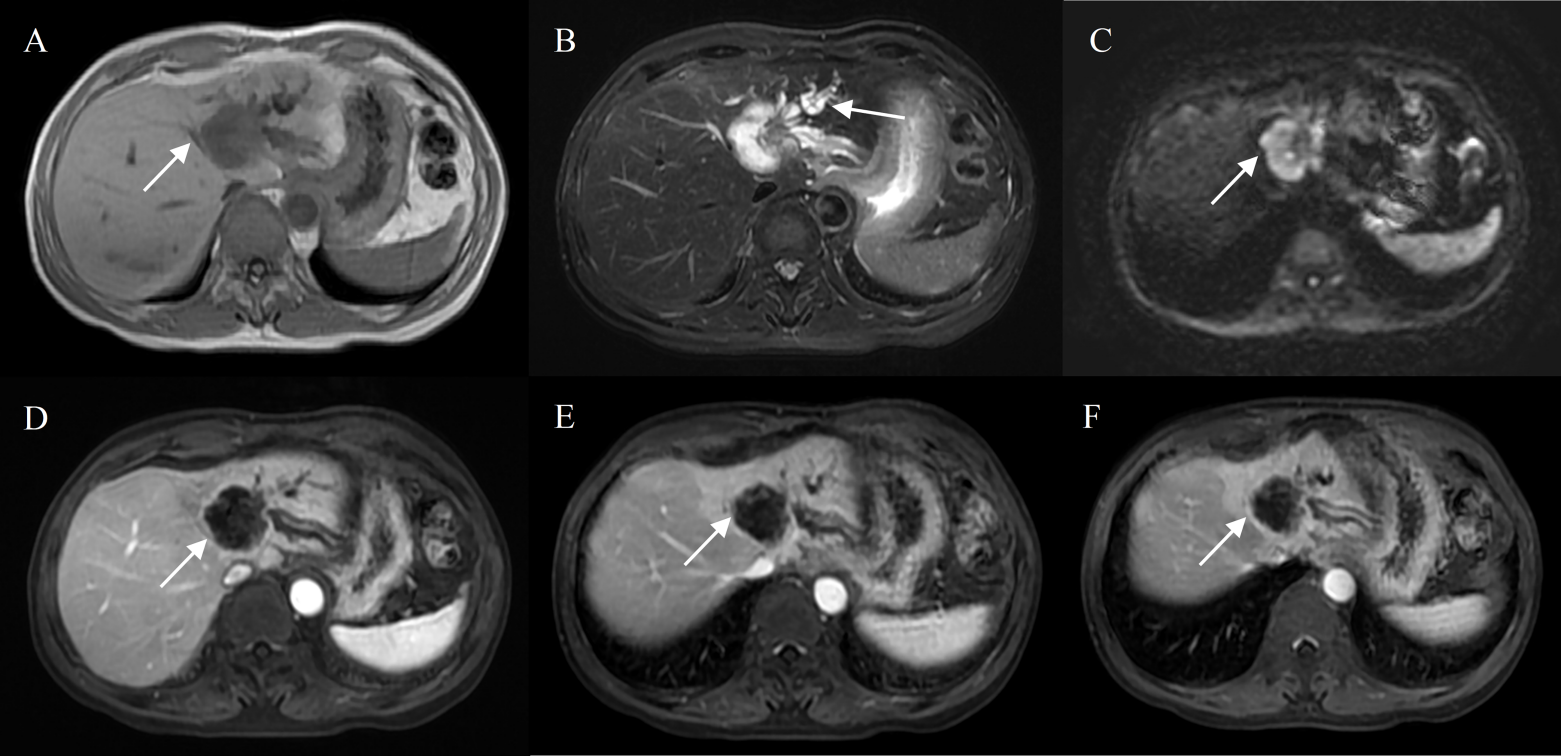
Intrahepatic mass-forming cholangiocarcinoma in a 70-year-old male without chronic viral hepatitis. Gd-DTPA-enhanced MRI detected a lobulated tumor (4.3 cm) with homogeneous SI on T1WI and T2WI **(A, B)**, restricted diffusion **(C)**. Peripheral rim-like enhancement in the arterial phase **(D)**, sustained rim enhancement in the portal and delayed phase **(E, F)**. And bile duct dilation peripheral to the tumor **(B)**.

Bile duct dilation (P<0.001), surface retraction (P<0.001), target sign on DWI (P = 0.002), and lymph node enlargement (P = 0.013) were significant imaging features of IMCCs. Radiological capsule (P<0.001), intralesional fat (P<0.001), central brightness on T2WI (P<0.001), and portal vein embolus (P<0.001) were significant imaging features of HCCs (**Table 2**). The representative images of atypical HCCs with rim-like enhancement cases are displayed in **Figures 2** and **3**, and IMCC cases are displayed in **Figures 4** and **5**.

### 3.3 Univariate and multivariate analysis of independent differences between HCCs and IMCCs

In univariate analysis, 17 features were significantly different between the two groups at a test level of P<0.05 (**Table 3**). All of the above 17 variables were included in multivariate logistic regression analysis (forward LR), which determined that radiological capsule (odds ratio (OR) 0.024, 95% confidence interval (CI): 0.006, 0.095, P<0.001), heterogeneous SI on T1WI (OR 0.009, 95%CI: 0.001,0.056, P<0.001) were independent differential factors for predicting HCC over IMCC; a lobulated contour (OR 11.732, 95%CI: 2.928,47.007, P = 0.001), target sign on DP (OR 14.269, 95%CI: 2.849,82.106, P = 0.007), bile duct dilatation (OR 12.856, 95%CI: 2.013, P = 0.001) were independent differential factors for predicting IMCC over HCC (**Table 4**).

**Table 3:** Univariate analysis for distinguishing HCCs from IMCCs.

Variable	OR	95%CI	P Value
Age	0.958	0.929, 0.988	0.006
Sex(F)	3.244	1.483, 7.092	0.003
Liver disease	0.048	0.020, 0.118	<0.001
Largest diameter	0.746	0.657, 0.847	<0.001
Shape (Lobulated)	3.905	1.971, 7.737	<0.001
Heterogeneous SI on T2WI	0.206	0.106, 0.399	<0.001
Heterogeneous SI on T1WI	0.096	0.044, 0.211	<0.001
Thin rim on AP	4.809	2.234, 10.351	<0.001
SI on DP			0.004
Target	4.330	1.820, 10.3	0.001
Iso	4.725	1.174, 19.02	0.029
Gradual enhancement	6.644	3.383, 13.047	<0.001
Surface retraction	6.859	2.24, 21.005	0.001
Bile duct dilatation	9.763	3.237, 29.446	<0.001
Intrallesional fat	0.099	0.022, 0.443	0.002
Central brightness on T2WI	0.260	0.129, 0.526	<0.001
Radiological capsule	0.047	0.021, 0.104	<0.001
Target on DWI	2.245	1.196, 4.211	0.012
Lymph node enlargement	3.421	1.527, 7.664	0.003

OR, odds ratio; CI, confidence interval. Variables with an odds ratio (OR) higher than 1.0 suggest IMCC, and variables with an OR lower than 1.0 suggest HCC.

Abbreviations can be found in the notes of **Table 2**.

**Table 4:** Multivariate analysis for distinguishing HCCs and IMCCs.

Variable	OR	95%CI	P Value
Radiological capsule	0.024	0.006, 0.095	<0.001
Heterogeneous SI onT1WI	0.009	0.001, 0.056	<0.001
Shape (Lobulated)	11.732	2.928, 47.007	0.001
SI on DP			
Target	14.269	2.849, 71.474	0.001
Iso	4.039	0.42, 38.807	0.227
Bile duct dilatation	12.856	2.013, 82.106	0.007

Variables with an odds ratio (OR) higher than 1.0 suggest IMCC, and variables with an OR lower than 1.0 suggest HCC.

Abbreviations can be found in the notes of **Tables 2** and **3**.

### 3.4 Model development, evaluation and visualization

**Figure 6:**
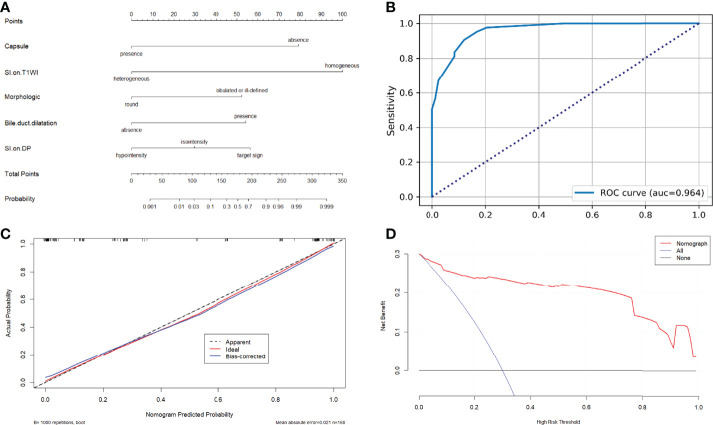
**(A)** Nomograms for distinguishing atypical HCCs with peripheral rim-like enhancement from IMCCs. **(B)** The receiver operating characteristic curve for the model. **(C)** The calibration curve analysis for the model. **(D)** The decision curve analysis (DCA) for the model.

A nomogram based on the model for distinguishing HCCs and IMCCs is shown in **Figure 6A**. The area under the ROC curve (AUC), sensitivity, and specificity values for the model were 0.964(0.940,0.987), 0.880, and 0.906, respectively (**Figure 6B**). It was further evaluated using calibration curves (**Figure 6C**), which showed that the discriminated HCC probability from the nomogram is consistent with the estimated value of the actual HCC probability. The model’s DCA curve showed an excellent net clinical benefit (**Figure 6D**). The results demonstrated that the model had an excellent differential diagnosis performance. And two radiologists used the model to diagnose respectively. The area AUC, sensitivity, and specificity values were as follows: 0.943(0.909,0.976), 0.867, and 0.918 for reviewer1; 0.936(0.897,0.975), 0.880, and 0.906 for reviewer2.

## 4 Discussion

The distinction between HCCs and IMCCs is crucial for clinicians because the prognosis and treatment options differ considerably between the two types (19). Thus, a noninvasive way to distinguish iCCA and HCC preoperatively is needed (27). Nowadays, contrast-enhanced MRI is widely used for preoperative diagnoses and evaluation of liver tumors. However, around 40% of HCCs show atypical imaging features such as arterial phase hypovascularity or peripheral rim-like enhancement, which pose a significant diagnostic challenge for radiologists (14, 15). Therefore, the present study aimed to identify reliable imaging features to help accurately differentiate atypical HCC with rim-like enhancement from IMCC.

The study results demonstrated that the radiological capsule, heterogeneous SI on T1WI, a lobulated contour, target sign on DP, and bile duct dilatation were independent differential factors. Combining these independent differential factors established the model for discriminating HCCs from IMCCs. Based on previous research and expert advice, we screened out five independent differential factors to build a model for the simplicity of the model. By verifying and evaluating the model, the model achieved high sensitivity (0.88) and specificity (0.906), indicating that these imaging features could obtain an excellent differential diagnostic performance for distinguishing atypical HCCs with peripheral rim-like enhancement from IMCCs.

In our study, compared to IMCCs, HCCs with peripheral rim-like enhancement more frequently showed heterogeneous SI on T1WI or T2WI, which may be a result of central necrosis and ischemia, or fibrotic component of the tumor. In previous studies, the rim enhancement of the tumor was attributed to the amount of fibrotic component within the tumor or the central necrosis/ischemia of more aggressive tumors (15, 28), which was consistent with our research.

Furthermore, the present study detected the capsule in 81 HCCs (95.3%) and three IMCCs (3.6%) on pathological examination. Histologically, the HCC capsule consists of an inner layer that contains fibrous fibers, followed by an outer layer that contains portal venules (or sinusoids) and newly-formed bile ducts (24, 29). Capsule appearance is characteristic of HCC and attributed to tumor growth (25). For this reason, LI-RADS uses capsule appearance as a major imaging feature of HCC. Previous study reported that enhanced “capsule” was a reliable imaging feature to help identify HCC (30). Consistent with previous studies, on imaging analysis, the capsule was more common in HCCs (69,81.2%) than in ICCs (14,16.9%) on Gd-DTPA-enhanced MRI. These results demonstrated the excellent differential diagnostic value of the capsule on imaging.

In the present study, a lobulated contour, target sign on DP, and bile duct dilatation were independent differential factors for predicting IMCC over HCC. A lobulated contour was more common in IMCCs than in HCCs, which was a vital imaging feature of IMCCs. The target sign on DP might be attributable to necrosis/ischemia in tumors or central fibrosis, and peripheral tumor cell components sustained enhancement (16). In addition, bile duct dilatation also was an independent differential factor in distinguishing IMCCs and HCCs. This is mainly because IMCCs originates from the epithelial lining of the intrahepatic bile duct, so IMCCs can occlude intrahepatic bile duct and cause peripheral bile duct dilatation and cholangitis (31, 32).

Our data revealed that approximately 34.9% (29/83) of IMCCs were found in underlying chronic viral hepatitis, and 13.3% (11/83) patients with IMCCs had microscopic cirrhosis. Even though most IMCCs developed in normal liver, several risk factors have been reported, including chronic viral hepatitis and cirrhosis (33, 34).Moreover, previous studies demonstrated that large HCCs might show atypical enhancement features such as lack or weak arterial or rim-like enhancement (35, 36). We found that the tumor size of HCCs (7.8cm (5.8,10.2)) was larger than IMCCs size (5.6cm (4.1,7.0)) in our study, which was consistent with previous studies.

Previous studies reported that the target sigh in hepatobiliary phase of gadoxetic acid disodium enhanced MRI is a valuable imaging feature to differentiate IMCC from atypical HCC (37, 38). In cirrhotic patients, Hepatobiliary-specific agent enhanced MRI can effectively differentiate small HCC from recurrent nodules, and the hepatobiliary phase has a high diagnostic value for small HCC (39, 40). In the present study, we identified reliable imaging features on Gd-DTPA-enhanced MRI for distinguishing atypical HCCs with peripheral rim-like enhancement from IMCC. It follows that contrast-enhanced MRI remains an effective method for the preoperative diagnosis and evaluation of liver tumors.

The study has several limitations. First, the retrospective single-center nature of the survey might have introduced selection biases. Second, the amount of case is small and we did not conduct external validation. Third, the use of this kind of model is not easy in daily clinical practice, and we will be committed to better applying the research results to clinical practice in the future. Besides, our study only focused on histopathological and MR imaging features. In the future, we will further investigate clinical laboratory indicators to differentiate atypical HCCs from IMCCs.

In conclusion, in the present study, we identified reliable imaging features, including capsule, heterogeneous SI on T1WI, a lobulated contour, target sign on DP, and bile duct dilatation on Gd-DTPA-enhanced MRI, which was helpful for distinguishing atypical HCCs with peripheral rim-like enhancement from IMCC. Our findings may help radiologists better to differentiate HCCs with atypical enhancement patterns from IMCCs, thereby providing clinicians with more accurate preoperative imaging diagnoses to select appropriate treatment options.

## Data availability statement

The original contributions presented in the study are included in the article/**Supplementary Material**. Further inquiries can be directed to the corresponding authors.

## Ethics statement

This study was approved by the ethics committee of eastern hepatobiliary surgery hospital, The Third Affiliated Hospital of Shanghai Naval Military Medical University, China, and waived the requirement of obtaining written informed consent. Written informed consent for participation was not required for this study in accordance with the national legislation and the institutional requirements.

## Author contributions

SZ, LH, and NJ conceived the project and designed the study. SZ, LH, YF, YL, JZ, and YW performed the data extraction and collection. WL and SZ performed the data analysis. SZ, WL, and NJ wrote and revised the manuscript. All authors contributed to the article and approved the submitted version.

## Conflict of interest

The authors declare that the research was conducted in the absence of any commercial or financial relationships that could be construed as a potential conflict of interest.

## Publisher’s note

All claims expressed in this article are solely those of the authors and do not necessarily represent those of their affiliated organizations, or those of the publisher, the editors and the reviewers. Any product that may be evaluated in this article, or claim that may be made by its manufacturer, is not guaranteed or endorsed by the publisher.
