# Supplementary material for: Preoperative differentiation of hepatocellular carcinoma with peripheral rim-like enhancement from intrahepatic mass-forming cholangiocarcinoma on contrast-enhanced MRI
Source: Front Oncol. 2022 Nov 23;12:986713. doi: 10.3389/fonc.2022.986713 (PMC9726747; doi:10.3389/fonc.2022.986713)
Supplement: Supplementary file 1 [file DataSheet_1.docx]

***Supplementary Material***

**Supplementary Table 1. Detailed scanner and scan parameters.**

| **Sequences** | **Image plane** | **TR/TE (msec)** | **FOV (mm)** | **Flip angle** | **Thickness (mm)** | **Matrix** | **Scanning order** |
| --- | --- | --- | --- | --- | --- | --- | --- |
| FIESTA | C | 3.5/1.5 | 420 × 420 | 60 | 6 | 160 × 224 | 1 |
| T1WI | A | 190/4.3(2) | 420 × 420 | 80 | 6 | 256 × 160 | 3 |
| T2WI | A | 6667/85 | 420 × 420 | 160 | 6 | 320 × 224 | 4 |
| LAVA | A | 3.7/1.7 | 420 × 420 | 15 | 2.5 | 256 × 192 | 5 |
| 2D MRCP | Oblique | 4000/847 | 320 × 320 | 160 | 50 | 320 × 256 | 6 |

TR, repetition time; TE, echo time; FOV, field of View; T1WI, T1-weighted image; T2WI, T2-

weighted image; MRCP, magnetic resonance cholangiopancreatography.

**Supplementary Table 2. Inter-observer variability for each imaging feature**

| **Characteristic** | **Kappa value** |
| --- | --- |
| **Gradual MRI features** |  |
| Shape | 0.79 |
| Margin | 0.79 |
| SI on T1WI | 0.75 |
| SI on T2WI | 0.76 |
| **Enhancement MRI features** |  |
| AP enhancement | 0.77 |
| SI on PVP | 0.76 |
| SI on DP | 0.81 |
| Gradual enhancement | 0.80 |
| **Ancillary features** |  |
| Surface retraction | 0.76 |
| Bile duct dilation | 0.80 |
| Radiological capsule | 0.81 |
| Arterial peritumoral enhancement | 0.51 |
| Intralesional fat | 0.78 |
| Intratumor necrosis | 0.55 |
| Central brightness on T1WI | 0.39 |
| High SI on FS- T1WI | 0.54 |
| Central brightness on T2WI | 0.76 |
| Central darkness on T2WI | 0.77 |
| Target sign on DWI | 0.81 |
| Septum | 0.49 |
| Portal vein embolus | 0.81 |
| Lymph node enlargement | 0.79 |
